# Exceptional fracture resistance of ultrathin metallic glass films due to an intrinsic size effect

**DOI:** 10.1038/s41598-019-44384-z

**Published:** 2019-06-04

**Authors:** Oleksandr Glushko, Marlene Mühlbacher, Christoph Gammer, Megan J. Cordill, Christian Mitterer, Jürgen Eckert

**Affiliations:** 1 Erich Schmid Institute of Materials Science, Austrian Academy of Sciences, Montanuniversität Leoben, Jahnstrasse 12, 8700 Leoben, Austria; 2 Department of Materials Physics, Montanuniversität Leoben, Jahnstrasse 12, 8700 Leoben, Austria; 3 Department of Physical Metallurgy and Materials Testing, Montanuniversität Leoben, Franz-Josef-Strasse 18, 8700 Leoben, Austria; 4 Infineon Technologies Austria AG, Siemensstraße 2, 9500 Villach, Austria

**Keywords:** Surfaces, interfaces and thin films, Structural properties, Mechanical properties

## Abstract

Metallic glasses typically fail in a brittle manner through shear band propagation but can exhibit significant ductility when the sample size is reduced below a few hundreds of nanometers. To date the size effect was mainly demonstrated for free-standing samples and the role of extrinsic setup parameters on the observed behavior is still under debate. Therefore, in the present work we investigated the mechanical properties of polymer-supported sputtered amorphous Pd_82_Si_18_ thin films with various thicknesses. We show that the films exhibit brittle fracture for thicknesses far below 100 nm. A pronounced size effect resulting in extended crack-free deformation up to 6% strain was observed only in films as thin as 7 nm – a thickness which is lower than the typical shear band thickness. This size effect results in exceptional cyclic reliability of ultrathin metallic glass films which can sustain cyclic strains of 3% up to at least 30,000 cycles without any indication of fatigue damage or electrical conductivity degradation. Since the enhancement of mechanical properties is observed at ambient conditions using inexpensive substrates and an industrially scalable sputter deposition technique, a new research avenue for utilization of ultrathin metallic glasses in microelectronics, flexible electronics or nanoelectromechanical devices is opened up.

## Introduction

Amorphous metallic alloys, which are commonly called metallic glasses (MGs), possess a number of outstanding properties such as a high elastic limit and strength^1–3^, good corrosion and wear resistance^4–6^, excellent diffusion barrier properties^7,8^, as well as good biocompatibility or even antimicrobial effect^9–11^. However, the application of MGs as structural or functional materials is hindered due to their intrinsic brittleness which is manifested by a catastrophic fracture through propagation of highly localized shear deformation within shear bands^2,3^. Recently, a number of experimental investigations demonstrated that considerable ductility can be observed in MGs if the sample size is reduced below a critical value, typically on the order of a few hundred nanometers^12^.

The first demonstration of ductile deformation of nanoscaled MGs was provided by Guo *et al*. in 2007^13^. By means of *in-situ* tensile tests performed in a transmission electron microscope (TEM) it was shown that MG samples with a cross-sectional size of about 100 nm are able to deform in a ductile manner and fail at large plastic strains through the formation of a neck instead of a shear band. Within the last decade the existence of ductile necking in nanoscaled MGs during tensile straining was confirmed by a number of other *in-situ* TEM investigations^14–21^. A similar size effect was also observed in micro-compression experiments^22–26^. Despite the significant number of experimental works reporting the size effect on the deformation behavior, the results reported so far are generally divergent and, in some cases, even controversial. For instance, the values of the critical size at which the transition to homogeneous deformation occurs span between 80 nm^17^ and 500 nm^16^. At the same time, there are several studies where this transition was not observed at all^27–30^. It was also shown that the size effect can occur in compression but is not observed under tension for the same MG composition^20^.

The large divergence of the reported experimental results can be partially explained by the large number of sample and setup parameters which could influence the measured mechanical properties. In particular, it was argued that extrinsic parameters such as the shape of tensile or compression samples^2,5,12,15–17^, machine stiffness^2,18,25,26^, implantation of Ga ions from focused ion beam (FIB) milling^12,15,17,22,31^, and electron beam irradiation^15–17,19^ can influence the observed mechanical behaviour. It is evident that it is virtually impossible to exclude all these external factors in the framework of a micromechanical test. Thus, input obtained from an alternative experimental method is of great importance.

Here, we consider polymer-supported amorphous Pd_82_Si_18_ thin films with thicknesses of 250, 100, 60, 16, 9, and 7 nm and lateral dimensions in the mm range. The films were deposited by unbalanced magnetron co-sputtering on polyimide substrates and were strained in tension to 10% under ambient conditions and without any additional sample preparation procedures such as grinding or FIB milling (see supplementary material for more details on film synthesis and characterisation). The structural integrity of the films during straining was monitored by *in-situ* measurements of electric resistance. This experimental design provides completely different test conditions in comparison to the micromechanical experiments^13–30^.

## Results and Discussion

### Glassy state of Pd_82_Si_18_ films

**Figure 1:**
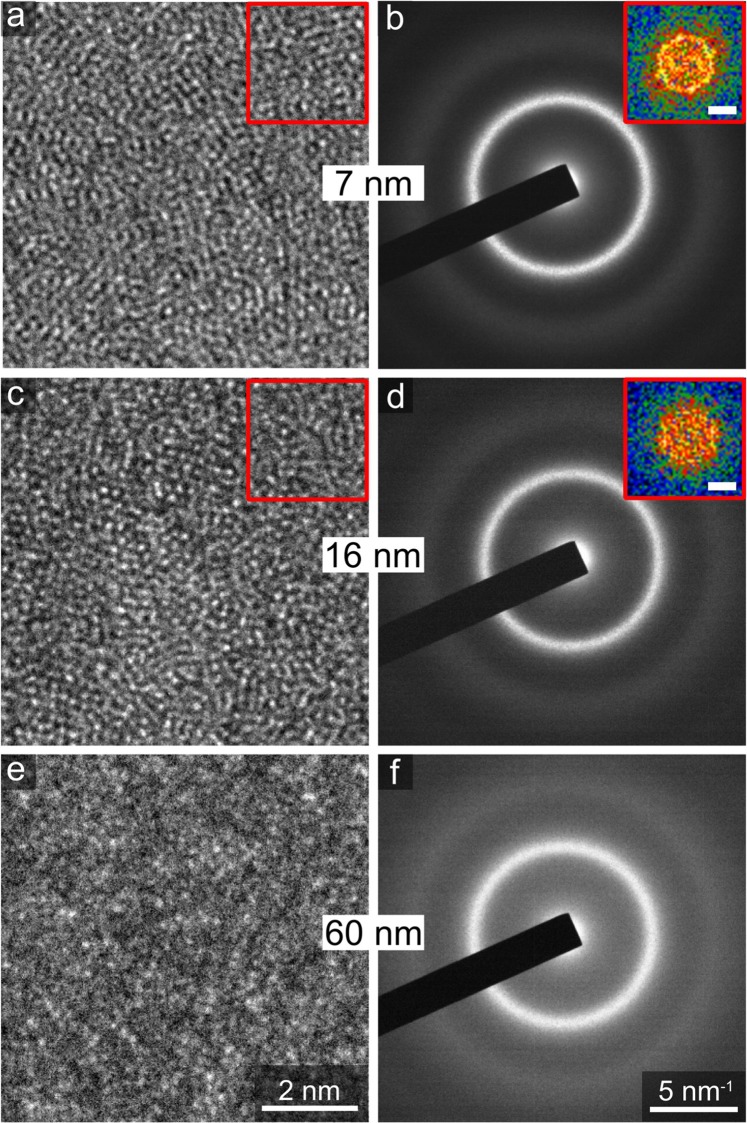
TEM verification of the glassy state of as-deposited films. HRTEM (**a,c,e**) and SAED (**b,d,f**) images of Pd_82_Si_18_ thin film MGs with thicknesses of 7, 16 and 60 nm, respectively. Both HRTEM and SAED images reveal a homogenous amorphous structure. The higher background observed for the 60 nm thick specimen is due to increased inelastic scattering. The insets in (**b**) and (**d**) shows the Fourier transforms obtained from the corresponding areas depicted by a red square in (**a**) and (**c**), respectively. The white bar in the insets corresponds to 5 nm^−1^.

The amorphous structure of the as-deposited Pd_82_Si_18_ films was confirmed by X-ray diffraction (XRD) and transmission electron microscopy (TEM) investigations. The XRD patterns of 250, 100, 60, 16, and 9 nm thick films are depicted in Supplementary Fig. S1. While the thicker films exhibit a broad peak characteristic for the amorphous phase, the diffraction maximum was too weak to be observed for film thicknesses below 60 nm despite the grazing incidence geometry. The position of the peak maximum shifts slightly from 2θ_max_ = 40.40° ± 0.04° for 250 nm thick films to 2θ_max_ = 40.28° ± 0.07° for 60 nm thick films. Although such a small peak shift is on the limits of statistical significance, it might indicate a slight increase in free volume with decreasing film thickness. Additionally, TEM investigations were carried out to capture possible structural differences in the ultrathin films. Typical high-resolution (HR)TEM images and corresponding selected area electron diffraction (SAED) patterns of 60, 16, and 7 nm thick films, which were deposited directly on TEM grids, are depicted in Fig. 1. Both, the HRTEM images and SAED patterns reveal that the samples are amorphous without presence of nanocrystals. The insets in Fig. 1a,c show diffractograms obtained through a Fourier transform of the sections marked by a red square on the corresponding HRTEM images. They show speckles but no clear crystalline reflections. Although these speckles can indicate the existence of some local ordering in the form of medium-range order in metallic glasses, it is hard to quantify it from HRTEM images due to the imaging artefacts caused by objective lens band pass filtering^32^. For interpretation of the results of mechanical testing shown below it is crucial to detect possible structural difference between 16 nm and 7 nm thick films since these two film thicknesses show dramatic difference in tensile behaviour. The diffraction profiles obtained from the SAED patterns reveal no significant difference in position and shape of the first amorphous diffraction maximum of 7 and 16 nm thick films (Supplementary Fig. S2), indicating that there is no significant difference in free volume^33^ or short-range order.

### Tensile behaviour

**Figure 2:**
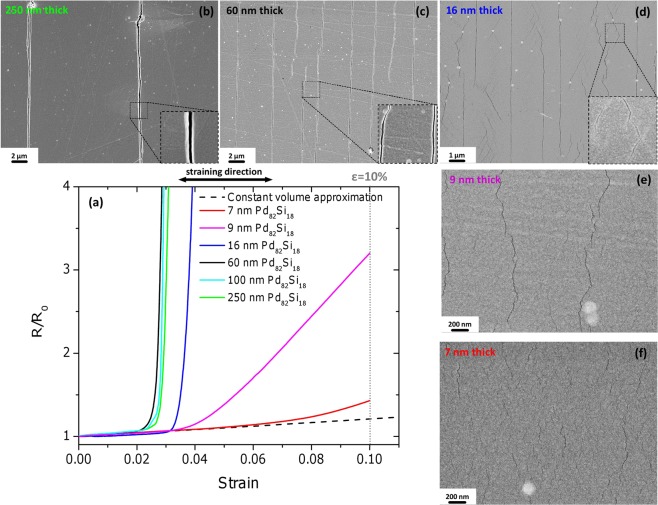
Tensile behaviour of sputter deposited Pd_82_Si_18_ metallic glass films on polyimide substrates. (**a**) Normalised electric resistance versus strain recorded *in-situ* during straining. The dashed line corresponds to the constant volume approximation. (**b**) through (**f**) are representative *post-mortem* SEM micrographs of 250, 60, 16, 9, and 7 nm thick PdSi films, respectively.

Tensile testing with *in-situ* measurements of electrical resistance is a well-established technique which enables exact determination of the critical strain at which cracks start to form in an electrically conductive film^34,35^. The crack onset strain (COS) can be clearly defined for brittle fracture and is indicated by the rapid growth of electric resistance^34^. Formation of cracks in ductile materials is usually manifested by a deviation of the measured resistance curve from the constant volume approximation curve, which shows how the resistance would increase if perfect homogeneous deformation (without cracking) occurred^35^. The evolution of the electrical resistance of Pd_82_Si_18_ films during monotonic tensile straining to 10% strain is shown in Fig. 2. The films with thicknesses of 250, 100, and 60 nm behave similarly with a COS of about 2%. The subsequent rapid growth of the electrical resistance in these films is attributed to the generation and propagation of brittle-like cracks, which are visualised in *post-mortem* scanning electron microscopy (SEM) images for 250 nm thick films (Fig. 2b) and 60 nm thick films (Fig. 2c). According to the shear lag model^36,37^ the saturation crack density induced by monotonic tensile loading is inversely proportional to the film thickness. This explains significantly lower crack density in 250 nm thick films (Fig. 2b) in comparison to 60 nm thick films (Fig. 2c). Electrical resistance at maximum strain (Supplementary Fig. S3) demonstrates the same trend for the three film thicknesses: 60 nm films have the highest resistance at 10% strain and 250 nm thick films – the lowest. The 16 nm thick films have a higher COS value of about 3%, but again very rapid growth of the resistance thereafter. The *post-mortem* SEM micrograph shown in Fig. 2d reveals a change in fracture morphology. There are two different types of cracks in the 16 nm thick films: long brittle-like cracks similar to those observed in thicker films and short cracks with irregular shape (inset in Fig. 2d). Due to the significant fraction of short cracks also the resistance at maximum strain (Supplementary Fig. S3) in 16 nm thick films is lower than in thicker films. The resistance of the 9 nm thick films starts to deviate from the constant volume approximation at 3.5% strain and increases only by a factor of 3 up to 10% strain. Such resistance behaviour corresponds to the formation of rather short and isolated cracks, as confirmed by the corresponding *post-mortem* SEM image (Fig. 2e). Finally, the resistance of 7 nm thick films follows the constant volume approximation up to a strain of about 6% and exhibits a very slow increase afterwards. The cracks, which can be seen only at relatively high magnifications, are homogeneously distributed through the whole film and they are only a few hundred nanometres long.

The COS value of 2% which is observed for 60, 100, and 250 nm thick films corresponds very well to the elastic limit of bulk metallic glasses^38^. One can thus conclude that these films start to rupture as soon as the elastic limit is reached. The 16 nm thick films demonstrate higher elastic strain of about 3% which is further increased to 3.5% for 9 nm thick films. The amount of elastic strain in the deformation of 7 nm thick films can be hardly determined within the current experimental approach. The absence of cracks up to the strain of 6% could be caused by both extended elastic deformation and distributed plastic deformation. An enhanced elastic limit was also observed for nanoscaled tensile MG samples with *in-situ* TEM experiments. An elastic strain as high as 6.6% was reported for free-standing amorphous NiNb films^39^ and elastic strains up to 5% were observed in amorphous CuZr nanotensile specimens^18,40^ all having sample dimensions over 50 nm.

### Film cross-sections and deformation mechanisms

**Figure 3:**
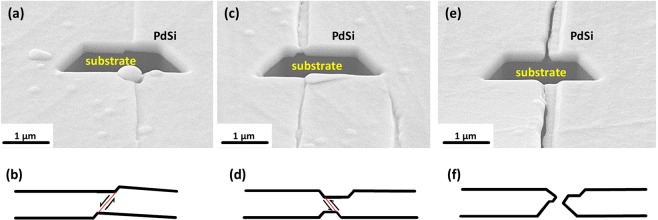
Post-mortem characterization of film cross-sections by FIB. Three different stages of crack/shear band evolution in 250 nm thick polymer-supported Pd_82_Si_18_ films after straining to 10% can be clearly distinguished. (**a**) Early stage deformation where a single shear event within a single shear band leads to the formation of a single step on the surface as explained in the schematic diagram (**b**). (**c**) An intermediate stage with a neck formed due to the activation of another shear band, as represented in (**d**). (**e**) A fully developed and open crack, the corresponding schematic diagram with the profile of crack edges is depicted in (**f**).

To elucidate the deformation and fracture mechanisms of polymer-supported MG films, the cross-sections of crack edges in 250 nm thick films were studied. Through the observation of a large number of different cracks it was possible to clearly identify three stages of crack evolution. The FIB cross-section of a 250 nm thick Pd_82_Si_18_ film demonstrating the early stage of crack formation, which is typically observed at the tip of a crack, is shown in Fig. 3a. A single step on the surface is formed through shearing within a single shear band which is depicted by a thin red line in the schematic diagram in Fig. 3b. A typical example of an intermediate stage of crack development is depicted in Fig. 3c, where a neck is formed through the activation of the second shear band oriented mirror-symmetric to the first shear band (Fig. 3d). It can be assumed that further shear slip events will occur parallel to the first or second shear band within this neck due to the reduced local cross-sectional area. A typical example of a fully developed open crack is shown in Fig. 3e. The schematic diagram for the corresponding profile of the crack edges is depicted in Fig. 3f. The cross-sections of 60 and 100 nm thick films exhibit features similar to that shown in Fig. 3. An example of the cross-section in 60 nm thick films is given in Supplementary Fig. S4.

Defining ductility as “*the ability to undergo plastic deformation before fracture*”, one can state that considerable local ductility is observed in 250 nm thick films as evidenced from Fig. 3. A simple argument proving that plastic deformation indeed occurred is that the crack edges do not match each other like two pieces of a puzzle, as would be expected for brittle cleavage or fracture through a single shear band. However, the observed local ductility cannot be attributed to a size effect since in the present case the film does not fail through only one single shear band because of the constraint provided by the substrate. Indeed, in the absence of delamination the shear displacement within a single shear band (Fig. 3a) will be inevitably stopped by the opposing force induced by the film-substrate adhesion and a mirror-symmetric shear band is activated to accommodate further tensile elongation. Thus, the ductility observed in 250 nm thick films is caused solely by the geometric constraint from the substrate and not by a size-dependent deformation mechanism. Despite the apparent similarity of the crack edge profiles to ductile necking (Fig. 3e), the deformation mechanism is not ductile necking but multiple slip within two groups of shear bands. The situation is different for the film thicknesses below 16 nm. We hypothesize that the change in deformation behaviour observed for 7 nm thick films is caused by a real intrinsic size effect which manifests itself as complete suppression of shear band formation.

Molecular dynamics simulations suggest that, for the nucleation of a shear band, elementary shear transformations have to be activated within some critical material volume, typically 10 to 20 nm in diameter^2,41,42^. Experimental measurements of shear band thicknesses give the same length scale range^43–45^. The critical film thickness of 16 nm, below which the suppression of brittle-like fracture is observed, corresponds well to these estimations. The most straightforward explanation of the suppressed brittle fracture in ultrathin films is that shear bands cannot form due to the lack of available material volume. It is important to mention that all considered films have macroscopic lateral dimensions and contain inevitable defects and irregularities caused by substrate surface imperfections as well as by the sputter deposition process itself. Examples of such defects can be easily recognized in Fig. 2b–f as bright spots of circular shape. Thus, the absence of shear banding and crack propagation in 7 nm thick films cannot be explained by the statistical probability of having a critical defect within the sample, as is often argued in micromechanical experiments^2,18,26^. Moreover, the extrinsic factors which can influence the mechanical behaviour in micromechanical experiments are excluded in our experiments *a priori*. There is no FIB damage, no sample shape effect, no electron beam effect, no machine stiffness effect, and no effect of grip or sample-punch interface. The only extrinsic factor is the constraint provided by the substrate which leads to local ductility, as shown in Fig. 3, as well as to a thickness dependence of the crack density as described by the shear lag model^36^. However, the substrate constraint can hardly explain the dramatic difference in fracture morphology between 16 and 7 nm thick films since all samples are affected the same way by the substrate.

### Cyclic reliability

**Figure 4:**
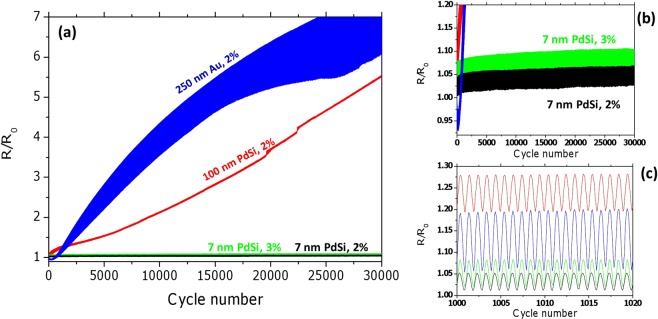
Evolution of electrical resistance of different polymer-supported films during cyclic tensile loading. (**a**) Overview comparing the 250 nm thick crystalline gold films to amorphous 100 nm thick PdSi film, and 7 nm thick PdSi films. (**b**) Enlarged portion of (**a**) demonstrating that virtually no resistance increase is observed in 7 nm PdSi films even for the cyclic strain of 3%. (**c**) Enlarged portion of (**a**) showing the fine structure of the recorded resistance signal.

Crack-free deformation of polymer-supported films is generally observed in ductile crystalline metal films with large enough grain size^35^. However, high ductility is a significant drawback if cyclic mechanical loading is applied. In crystalline metals formation of slip bands, extrusions and propagation of cracks is induced by very low cyclic strains typically far below 1%. The size effect in amorphous films provides the potential to obtain much higher fatigue strength due to the extended elastic strains and absence of dislocations. The behaviour of different thin films during cyclic loading is demonstrated in Fig. 4. For a cyclic strain of 2% the crystalline 250 nm thick gold film, shown for comparison, demonstrates fast growth of electrical resistance with cycle number that indicates propagation of fatigue cracks. The increase of the thickness of the curve corresponding to the 250 nm Au film is due to the increasing difference in resistance between zero and peak strain and can be explained by crack re-bridging during the unloading portion of each cycle^46^. Similarly, a cyclic strain of 2% induces significant cracking also in the 100 nm thick PdSi films as they exhibit a monotonic increase of resistance with increasing cycle number. In contrast, the 7 nm thick PdSi films show no change in resistance as can be clearly seen in Fig. 4b. Even a cyclic strain of 3% does not affect the measured resistance, which indicates that no through-thickness cracks are formed during cyclic loading.

### Potential alternative explanations of the observed behaviour

Although the observed drastic change in the cracking behaviour of ultrathin films can be consistently explained by the size effect, it is necessary to examine alternative models. The most plausible alternative explanation would be to assume a significantly different structure for the 7 nm thick films as compared to the thicker ones. The 7 nm thick films on polyimide could have been deposited in a rejuvenated state, i.e. having higher amount of free volume, which could lead to the extended crack-free deformation. We hold this alternative explanation as improbable due to the following reasons. First of all, a significant difference between the 16 nm thick film and 7 nm should be visible in the short range order which is not the case according to Supplementary Fig. S2. Secondly, the hypothesis of the existence of additional free volume in 7 nm thick film does not explain total suppression of crack propagation. For instance, Magagnosc *et al*.^31^ reported just a few per cent of increased tensile ductility due to addition of free volume in MG nanowires. Such a dramatic change in fracture morphology between 7 and 16 nm thick films as shown in Fig. 2 can be hardly attributed to subtle changes in free volume.

It is also important to note that in the case of brittle crystalline films the fracture strain can be increased by increasing the residual compressive stress in as-deposited state^47^. In this case, however, only the COS value is shifted without any change of the fracture morphology^47^. Besides, residual compressive stress in a metallic glass can be interpreted as more relaxed (or “aged”) state which must exhibit even more brittle behaviour^31^. Thus, extended crack-free deformation of 7 nm thick films cannot be attributed to some specific residual stress state of the film after deposition.

## Summary

To sum up the main implications of the presented work, first and foremost, a strong size effect in the deformation behaviour of metallic glasses is demonstrated in thin films with macroscopic lateral dimensions at ambient conditions. The 7 nm thick Pd_82_Si_18_ films exhibit crack-free deformation up to 6% strain and distributed initiation of isolated nanosized cracks afterwards. Secondly, local ductility and necking of thicker films (e.g. 250 nm thick) occurs through subsequent activation of differently oriented and localized shear bands. This pseudo-ductility is possible solely due to the constraint provided by the substrate and should not be interpreted as a size effect. The third implication is that, in contrast to the results of micro-mechanical experiments, the critical size for the transition to homogeneous deformation corresponds very well with theoretical and experimental estimations of the critical volume for the generation of a shear band. Therefore, we conclude that this is a real intrinsic size effect occurring due to suppression of shear banding as a deformation mechanism. It is important to note that the observed size effect is attributed to the fundamental deformation mechanisms of amorphous metallic alloys and not to a particular chemical composition. Consequently, it is expected that similar behaviour can be observed for a large variety of thin film MGs analogous to the elastic strain of about 2% which is a general attribute of MGs independent of the chemical composition^2,3^. However, further work is required to prove the generality of shear band suppression in films with thicknesses below the thickness of shear bands.

**Figure 5:**
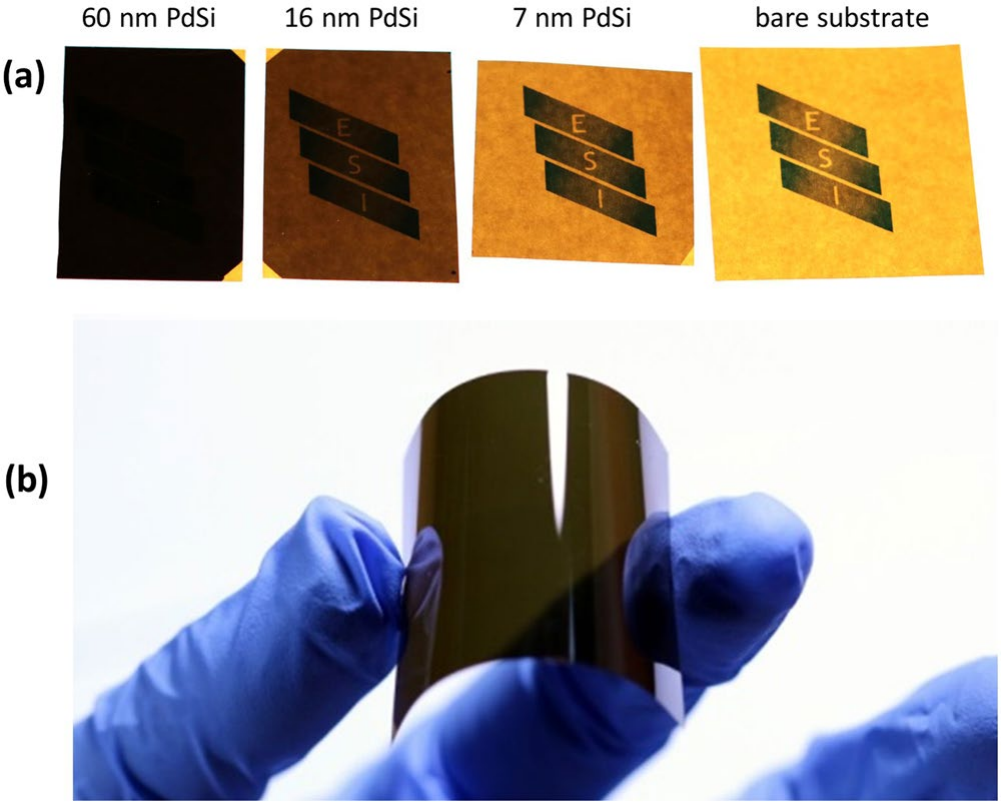
Demonstration of optical transparency (**a**) and mechanical flexibility (**b**) of amorphous Pd_82_Si_18_ films on polyimide substrates. The institutional logo printed on a white paper cannot be seen through fully opaque 60 nm thick film, is already recognizable through 16 nm thick film while 7 nm thick film is half-transparent.

Ultrathin film MGs on flexible substrates show a unique combination of properties. Apart from their exceptional fracture resistance stemming from the size effect, the ultrathin films are half-transparent (see Fig. 5) and have acceptable resistivity of the order of 100 µΩ-cm. Such a property combination make MG thin films suitable for transparent conductive coating applications as demonstrated in a comparative table given in the supplementary material (Supplementary Table 1). Finally, it should be noted that the samples were obtained using an industrially scalable deposition process on commercially available substrates and testing was carried out at ambient conditions. This makes ultrathin MGs directly applicable for microelectronics, flexible electronics and nanoelectromechanical systems^48^.

## Methods

Pd_0.82_Si_0.18_ thin films with thicknesses in the range of 7–250 nm were deposited in a custom-built laboratory-scale unbalanced dc magnetron sputtering system with a base pressure <10^−4^ Pa. The deposition system was fitted with two 2 inch diameter circular targets (Pd, 99.95% purity; Si, 99.999% purity) in a confocal arrangement. The substrate materials, polyimide Upilex® foil (50 × 50 mm^2^), Si(001) with a native oxide layer (20 × 7 mm^2^, B-doped, resistivity *ρ* = 1–20 Ω-cm), NaCl(001) (10 × 10 mm^2^), MgO(001) (10 × 10 mm^2^), and ultrathin TEM support grids were mounted on a rotatable sample holder opposite to the targets with a target-to-substrate distance of 40 mm. Si(001), MgO(001), and NaCl(001) substrates were cleaned in ultrasonic baths of acetone and ethanol and blown dry with dry air immediately prior to loading them into the vacuum system. The polyimide foil was not subjected to any wet cleaning, but dust particles were removed with pressurised air.

Prior to deposition, the targets were sputter cleaned in pure Ar for 30 sec, with shutters protecting the substrates and adjacent targets. The thin films were deposited in Ar atmosphere with pressure of 0.4 Pa. No intentional substrate heating was applied. The target power was set to 60 W at the Pd target and 140 W at the Si target, resulting in a deposition rate of 0.7 nm/s. Six different batches of films are discussed in the present study, with thicknesses of 250, 100, 60, 16, 9, and 7 nm.

The chemical composition of the films was determined by energy-dispersive X-ray spectroscopy (EDX) in a Zeiss Leo 1525 scanning electron microscope on the 250 nm-thick film grown on NaCl(001) and confirmed by EDX on a free-standing film floated from the NaCl(001) substrate in a Philips CM 12 TEM microscope operated at 120 kV.

### Film characterization

Film thicknesses were determined by surface step-height measurements using chromatic confocal profilometry. To this end, one side of a Si(001) substrate was masked with Kapton™-tape. After film deposition, the tape was removed to produce a sharp surface step. Profilometry was carried out over randomly-chosen areas of 0.5 × 0.5 mm^2^ across the step, using a Wyko NT 1000 optical three-dimensional white-light profiling system. The film thicknesses given for the 250, 100, and 60 nm films have an accuracy of ±5 nm, for thinner films the measurement accuracy is ± 1 nm.

Additional film thickness measurements were carried out by X-ray reflectivity (XRR) in a Rigaku SmartLab 5-axis diffractometer operated at 40 kV and 30 mA with Cu–K_α_ radiation parallelized in a parabolic multilayer mirror. The device was equipped with a 0.1 mm vertical slit, a double-bounce Ge(220) monochromator, and a 5.0 mm length limiting slit in the incident beam as well as 0.2 and 0.4 mm vertical slits and a 5.0° Soller slit in the scattered beam. The incidence angle Θ was increased from 0 to 3° in 0.005° steps. The collected XRR data were evaluated with the Python-based refinement program GenX [for details see M. Björck *et al*., *J. Appl. Crystallogr*. **40**, 1174 (2007)], utilizing the Parratt recursion formula for reflectivity simulation and the genetic differential evolution algorithm for data refinement. The comparison of film thicknesses measured by a profilometer and obtained from XRR measurements measured thicknesses is given in Supplementary Fig. S1.

X-ray thin film diffraction in grazing incidence (incidence angle 2°) geometry from 20° < 2Θ < 80° in 0.02° steps was performed in the Rigaku diffractometer described above. For these measurements the device was equipped with a 5.0° Soller slit, a 1.0 mm vertical slit and a 5.0 mm length limiting slit in the primary beam as well as a two 20.0 mm vertical slits, a 0.5° parallel beam analyser, a 5.0° Soller slit, and a flat graphite monochromator in the diffracted beam.

Samples for TEM investigations were obtained by depositing the thin film MG directly on ultrathin TEM support grids (single layer graphene and 3 nm thick carbon, Pelco). HRTEM images and SAED patterns were taken with a JEOL 2100 F microscope equipped with a spherical aberration corrector operated at 200 kV. The diffraction patterns were obtained using a selected area aperture with a diameter of 1 µm.

### Mechanical testing

Polymer-supported thin film MG samples with a width of 4 mm and a length of 40 mm were cut from larger sheets using a scalpel. Uniaxial tensile tests were performed on an MTS Tytron 250 universal tensile machine in displacement-controlled mode using an initial gauge length of 20 mm and a displacement rate of 5 µm/s. The electrical resistance was measured *in-situ* during straining in four-point probe geometry with the contacts incorporated directly in the grips. At least four samples of each type were tested. Cyclic loading experiments were performed by applying a sine strain function oscillating between the peak strain (2% or 3%) and zero strain with the frequency of 0.5 Hz.

### Post mortem characterization

Strained films were characterized using a Zeiss Leo 1525 scanning electron microscope. Focused ion beam cross-sectioning was performed on a dual beam Zeiss Leo 1540 workstation.

## Supplementary information

Supplementary information

## Data Availability

The data acquired in the course of this study are available from the corresponding author on request.
