# Supplementary material for: Exceptional fracture resistance of ultrathin metallic glass films due to an intrinsic size effect
Source: Sci Rep. 2019 Jun 4;9:8281. doi: 10.1038/s41598-019-44384-z (PMC6547732; doi:10.1038/s41598-019-44384-z)
Supplement: Supplementary file 1 — Supplementary information [file 41598_2019_44384_MOESM1_ESM.docx]

Supplementary Information

**Exceptional fracture resistance of ultrathin metallic glass films due to an intrinsic size effect.**

O. Glushko*^1^, M. Mühlbacher^2‡^, C. Gammer^1^, M. J. Cordill^1,2^, C. Mitterer^3^, J. Eckert^1,2^

*^1^Erich Schmid Institute of Materials Science, Austrian Academy of Sciences, Jahnstrasse 12, 8700 Leoben, Austria*

*^2^Department of Materials Physics, Montanuniversität Leoben, Jahnstrasse 12, 8700 Leoben, Austria*

*^3^Department of Physical Metallurgy and Materials Testing, Montanuniversität Leoben, Franz-Josef-Strasse 18, 8700 Leoben, Austria*

***corresponding author:** [**oleksandr.glushko@oeaw.ac.at**](mailto:oleksandr.glushko@oeaw.ac.at)

‡**current address:** Infineon Technologies Austria AG, Siemensstraße 2, 9500 Villach, Austria

**1. X-ray diffraction analysis**


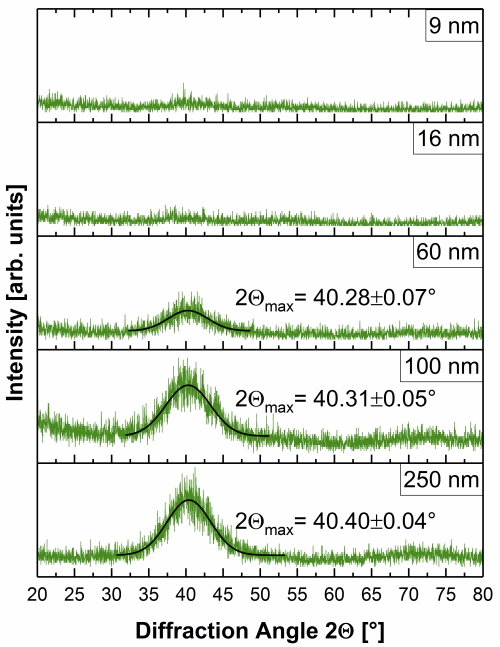


**Supplementary Figure S1.** X-ray diffractograms of Pd_82_Si_18_ thin film MGs exhibit no significant shift of the peak characteristic for the amorphous phase at 2*Θ_max_*, indicating no significant changes in the short range order with decreasing film thickness.

**2. TEM analysis**

**
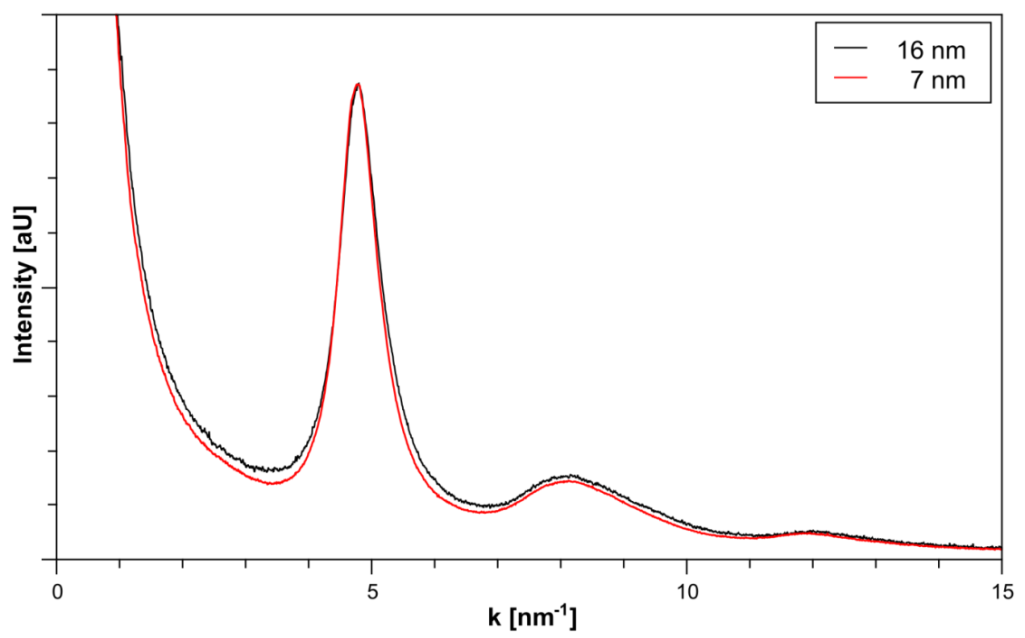
**

**Supplementary Figure S2.** Comparison between electron diffraction profiles of 16 and 7 nm thick Pd_82_Si_18_ thin films revealing no significant change in the short-range order.

**3. Full in-situ resistance curves.**

**Supplementary Figure S3.** The evolution of electrical resistance of the Pd_82_Si_18_ films plotted in logarithmic scale. The curves are identical to those shown in Fig. 2a. Clear correlation between the crack density and resistance growth at 10% strain is observed for 250, 100, and 60 nm thick films in accordance to the shear lag model. The thinner films (16 nm and below) exhibit a change in crack morphology leading to decreasing resistance growth values at 10% strain.

**4. Focused Ion Beam cross-section of 60 nm thick film.**


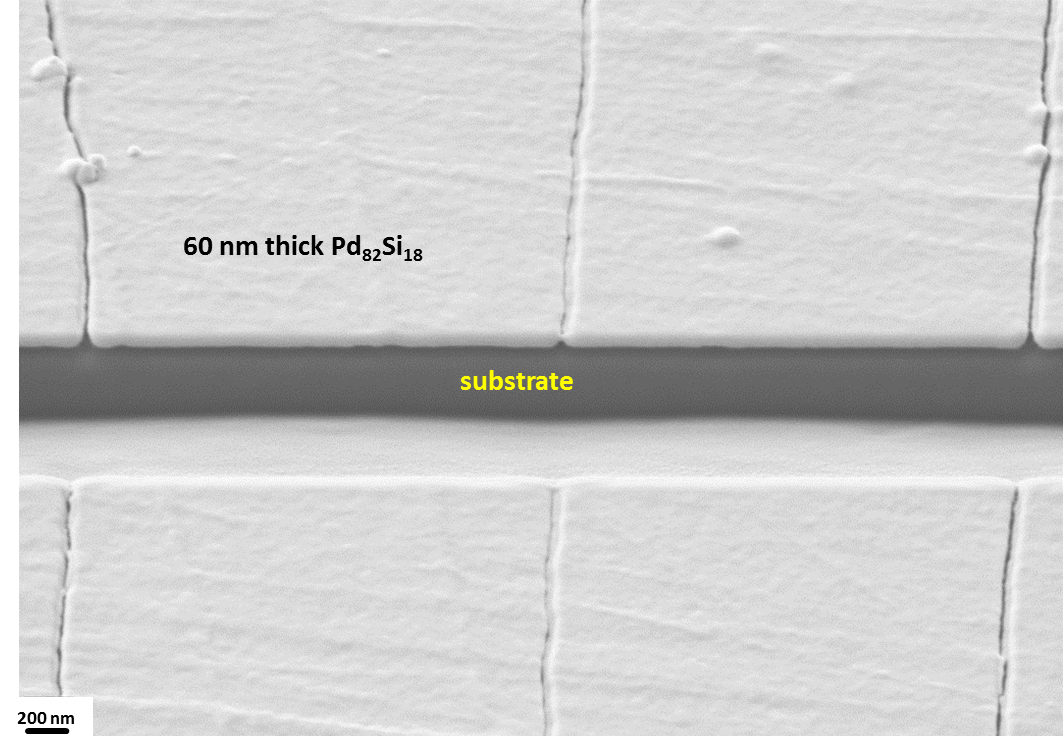


**Supplementary Figure S4.** FIB-milled cross-section in 60 nm thick PdSi film demonstrating the same local ductility mechanisms as shown in the paper Fig. 3.

**7. Comparative table of transparent conductive coatings**

| Material | Thickness/NWØ  [nm] | COS  [%] | Sheet Resistance  [Ω/□] | Transmittance  [%] | Ease of production |
| --- | --- | --- | --- | --- | --- |
| PdSi film | 7 | 6 | 97 | 66 | + |
| ITO | 100-200 | <2^1^ | 10-200^1,2^ | 85-90^1,2^ | + |
| Ag/Au film | 10-30 |  | 50-500^3^ | 30-60^3^ | + |
| Au/Ag NWs | 40-100 | ~2*^4^ | 10-200^3,5^**^,^**^6^ | 40-90^3,5^**^,^**^6^ | - |
| Ag NWs | 25 | ~4*^4^ |  |  | - |
| CNT | Single wall |  | 56^2^ | ~70^2^ | - |
| Graphene | Single layer | 0.6^7^ | 679^7^ | 98^7^ | - |
| Graphene | Multilayer | ~30#^7^ | 10-500^2,7^ | 80-92^2,7^ | - |

**Supplementary Table 1.** Comparison of semi-transparent materials for flexible electronics.

* from bending test, # cracking observed but only small increase in resistance.

References used in Supplementary Table 1:

1. Sierros, K. A., Morris, N. J., Ramji, K. & Cairns, D. R. Stress-corrosion cracking of indium tin oxide coated polyethylene terephthalate for flexible optoelectronic devices. *Thin Solid Films* **517,** 2590–2595 (2009).

2. De, S. & Coleman, J. N. Are There Fundamental Limitations on the Sheet Resistance and Transmittance of Thin Graphene Films? *ACS Nano* **4,** 2713–2720 (2010).

3. Angmo, D., Espinosa, N. & Krebs, F. Indium Tin Oxide-Free Polymer Solar Cells: Toward Commercial Reality. *Low-cost Nanomater.* 189–225 (2014). doi:10.1007/978-1-4471-6473-9

4. Kim, D. *et al.* Failure criterion of silver nanowire electrodes on a polymer substrate for highly flexible devices. *Sci. Rep.* **7,** (2017).

5. Lyons, P. E. *et al.* High-Performance Transparent Conductors from Networks of Gold Nanowires. *J. Phys. Chem. Lett.* **2,** 3058–3062 (2011).

6. Scardaci, V., Coull, R., Lyons, P. E., Rickard, D. & Coleman, J. N. Spray Deposition of Highly Transparent, Low-Resistance Networks of Silver Nanowires over Large Areas. *Small* **7,** 2621–2628 (2011).

7. Won, S. *et al.* Double-layer CVD graphene as stretchable transparent electrodes. *Nanoscale* **6,** 6057–6064 (2014).
